# Course and predictors of social security disability insurance in patients with borderline personality disorder over 24 years of prospective follow-up

**DOI:** 10.1186/s40479-023-00236-x

**Published:** 2023-10-09

**Authors:** Ueli Kramer, Christina M. Temes, Frances R. Frankenburg, Isabel V. Glass, Mary C. Zanarini

**Affiliations:** 1 Department of Psychiatry, Institute of Psychotherapy and General Psychiatry Service, University of Lausanne, Place Chauderon 18, CH-1003 Lausanne, Switzerland; 2 Massachusetts General Hospital, Harvard Medical School, Boston, USA; 3 Edith Nourse Rogers VA Medical Center, Boston University School of Medicine, Boston, USA; 4 McLean Hospital, Belmont, USA; 5 McLean Hospital, Harvard Medical School, Boston, USA

**Keywords:** Borderline personality disorder, Social security disability insurance, Long-term follow-up, Psychosocial functioning

## Abstract

**Background:**

The utilization of Social Security Disability Insurance (SSDI) is frequent in patients with borderline personality disorder (BPD) and may represent a meaningful marker of a patient’s symptom severity, poor psychosocial functioning, and/or inner suffering. Over 24 years of prospective follow-up, the present study aims to describe the course of SSDI and assess the role of clinically relevant predictors.

**Methods:**

A total of 290 inpatients with BPD were interviewed at baseline and 12 consecutive follow-up waves, each separated by two years, after index hospitalization. Included were also 72 inpatients with other personality disorders. Surviving patients were reinterviewed. A series of interviews and self-report measures were used to assess psychosocial functioning and treatment history, axis I and II disorders, and childhood/adult adversity.

**Results:**

Results show that rates of SSDI utilization were relatively stable over 24 years of follow-up (on average, 47.2% of the patients with BPD were on SSDI). Patients with BPD were three times more likely to be on SSDI than patients with other PDs. Patients with BPD displayed flexibility in their usage of SSDI. By 24 years, 46% of patients remitted, out of which 85% experienced recurrence and 50% of the patients had a new onset over time. In multivariate analyses, four variables were found to predict SSDI status in patients with BPD over time. These variables were: age 26 or older, lower IQ, severity of non-sexual childhood abuse, and presence of PTSD.

**Conclusions:**

The results of this study suggest that a combination of a demographic factors, childhood adversity, natural endowment, and comorbidity are significant predictors of receiving SSDI over time. On a group level, there is a relative stability of SSDI usage over time, but on the individual level, the present study found a high fluctuation in receiving SSDI over 24 months of prospective follow-up.

## Introduction

Only a few studies have described the course of Social Security Disability Insurance (SSDI) in borderline personality disorder (BPD). They all found high rates of SSDI utilization in patient populations with BPD (i.e., between 12 and 72%; [19, 22, 23]. Longitudinal studies reported between 22 and 47% of patients with BPD receive social security disability insurance over two to 10 years of follow-up after index inpatient treatment [18, 20, 31], or after intense partial hospitalization [21].

In the United States, all individuals who fall short of being self-supportive of their basic needs (by a regular occupation) are eligible to receive SSDI. In this context, SSDI is a federal program, as a subpart of Social Security that provides an income and makes people eligible for vouchers for housing, support to buy food for good nutrition, as well as access to both federal programs for health insurance (Medicare and Medicaid).

It appears that the high rates of SSDI utilization in patients with BPD may also contribute to the perception of a high burden of disease associated with the disorder [1, 24]. It was observed that patients with BPD tend to use substantial resources of the societal, general health, and mental health systems [1, 7, 24, 37]. Among patients with BPD, symptomatic remissions are more common and stable than psychosocial recovery, particularly vocational stability, which may lead to the potential for financial instability in this patient population. This may suggest an ongoing need for SSDI to ensure financial stability [6, 7, 31, 37].

Qualitative and mixed methods research focused on the patient’s subjective perspective on recovery and utilization of SSDI in patients with BPD. Larivière et al. [14, 16, 5] showed that the term of “recovery” was associated in patients with BPD with the subjective idea of “moving forward” in life, and of “acceptance”, but also of “functioning well”. The latter may be associated with the individual’s motivation to get off social security payments, as they may possibly be seen as interfering with a fulfilled working life by recovered individuals. Absence of recovery was subjectively associated with perceived pressure (i.e., interpersonal stress) and negative functioning in the workplace (i.e., poor communication). Juurlink et al. [10] studied barriers and facilitating factors to be employed using qualitative interviews: the informants with BPD evoked the features of their disorder (i.e., interpersonal difficulties), societal stigma and support for employment (or lack thereof) as key.

Compared with other personality disorders over ten years of follow-up, Zanarini et al. [41] found that patients with BPD were three times more likely to receive SSDI than axis II comparison patients. Utilization of SSDI is not static: these authors found that 40% of those who were on disability at baseline were able to get off at some point, and 43% of those patients eventually went back on SSDI. Also, over 10 years of follow-up, 39% of patients began to receive SSDI for the first time. This study has demonstrated that SSDI utilization might be more fluid than previously assumed. Despite the public health interest related to this question, there are currently no follow-up studies on patient populations over long periods of time to demonstrate the ongoing course of SSDI and its fluidity. Most importantly, it remains unclear what the baseline predictors are of the course of SSDI. In addition, the course of SSDI was examined over time periods of up to 10 years [41], but it remains unclear about the follow-up periods of up to 24 years of prospective assessment after baseline, in comparison with other personality disorders (see for such a comparison for the course of depression, [40].

The present study uses a prospective methodology describing the course of SSDI utilization over time, over 24 years of structured follow-up, for a study population with borderline personality disorder, in comparison with those with other personality disorders. Baseline variables were examined as potential predictors of SSDI over time. Knowing about potential risk and protective factors related to SSDI may bear important clinical implications; the former can be targeted and the latter supported in treatment.

## Methods

### Participants

The present study uses data from the McLean Study of Adult Development (MSAD), a prospective longitudinal study on the course of borderline personality disorder over 24 years. Baseline data were reported in earlier studies [32]. Inclusion criteria were age between 18 and 35, an IQ of 71 and higher, and absence of history or current symptoms of schizophrenia and other psychotic disorders, bipolar disorder I, and organic conditions that can cause serious psychiatric symptoms (e.g., lupus, multiple sclerosis). Included were patients with BPD vs. other personality disorders (OPD) at their hospitalization in psychiatry [32]. The project was reviewed and approved by the McLean Hospital institutional review board.

### Instruments and procedures

After the study procedures were explained, written informed consent was obtained from each participant. Each patient then met with a masters-level interviewer blind to the clinical diagnoses for an interview on psychosocial and treatment history and diagnostic assessment. Five semistructured interviews were administered. These interviews were: 1) the Background Information Schedule (BIS, which assesses demographics, psychosocial functioning, and treatment history [31, 33], 2) the Structured Clinical Interview for DSM-III-R Axis I Disorders (SCID-I,[25], 3) the Revised Diagnostic Interview for Borderlines (DIB-R,[39], 4) Structured Clinical Interview for DSM-III-R (SCID) I [25], and 5) the Diagnostic Interview for DSM-III-R Personality Disorders (DIPD-R,[30]. The inter-rater and test–retest reliability of the BIS [31, 33] and of the three diagnostic measures [29, 38] have been found to be good to excellent.

Childhood history of pathological and protective experiences and history of adult experiences of adversity were assessed using two separate semistructured interviews by a second rater blind to all previously collected information. Childhood experiences were assessed using the Revised Childhood Experiences Questionnaire [42] and adult experiences of adversity were assessed using the Abuse History Interview [35]. The inter-rater reliability of these two interviews has also been found to be good to excellent [31]. In addition, self-report measures with well-established psychometric properties assessing temperament and intelligence were administered: the NEO Five Factor Inventory [4] and the Shipley Institute of Living Scale [28].

### Definition of Social Security Disability Insurance

For the present study focusing on the utilization of social security programs over 24 years of follow-up, social security disability insurance (SSDI) data was available at all 12 follow up waves, in addition to baseline information that was collected using the BIS. SSDI status was collected as part of the BFI-R (Revised Borderline Follow-up Interview; [36], at each follow-up wave. Interviewers at follow-up were blind to baseline information. Utilization of SSDI was defined as a binary variable for each follow-up wave: 0 = not on SSDI, 1 = on SSDI.

### Statistical analyses

Statistical analyses assessed the outcome of SSDI as either absent or present (0/1) for each time point and group (BPD vs. OPD). For the prevalence analyses, the generalized estimating equations (GEE) approach was used to model the rate of SSDI over 24 years of follow-up in patients with BPD (vs. OPD), as well as the predictors of receiving SSDI over time. Kaplan–Meier Survival Analyses were conducted to determine rates of remission, recurrence, and new onsets over 24 years. Predictor analyses were used to analyze the impact of a set of variables.

All predictor variables were initially examined individually as bivariate predictors of SSDI over time. To select the most salient subset of predictors of receiving SSDI on the multivariate level, we entered all the significant (*p* < 005, 2-tailed) variables from the bivariate analyses simultaneously and followed a backward deletion procedure until all variables remaining in the multivariate analysis resulted in a statistically significant model at 2-tailed *p* < 0.01. Analyses were performed using Stata 16.1 [26].

## Results

Two hundred and ninety patients met both DIB-R and DSM-III-R criteria for BPD at study entry; seventy-two patients met criteria for other personality disorders (OPD). Out of the patients with BPD, 80.3% (N = 233) of the patients were female and 87.2% (N = 253) were Caucasian. The average age of the patients was 26.9 years (SD = 5.8), their mean socioeconomic status was 3.4 (SD = 1.5) (where 1 = highest and 5 = lowest), and their mean GAF score was 38.9 (SD = 7.5) (indicating major impairment in several areas, such as work or school, family relations, judgment, thinking, or mood).

In terms of continuing participation, 83% (N = 206/247) of surviving patients with BPD (17 died by suicide and 26 died of other causes) were re-interviewed at all 12 follow-up waves and 79% (N = 53/67) of surviving patients with OPD (one died by suicide and four died of other causes) were re-interviewed at all 12 follow-up waves.

**Fig. 1:**
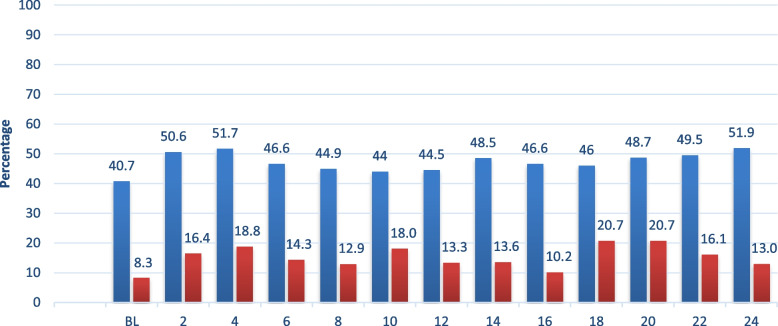
Prevalence of Social Security Disability Insurance (SSDI) in Patients with borderline personality disorder and other personality disorders over 24 years of prospective follow-up (%). *Note.* In blue prevalence of SSDI in patients with borderline personality disorder over time. In red prevalence of SSDI in patients with other personality disorders over time. SSDI: Social Security Disability Insurance

Figure 1 details the prevalence of being an SSDI recipient at each of our 13 assessment waves (baseline plus 12 follow-ups) for patients with borderline personality disorder (BPD) and other personality disorders (OPD). As can be seen, the rate for BPD is relatively stable over time, with a mean of 47.2%, as compared to a rather stable mean of 15.0% for other personality disorders. Those with BPD were 18% more likely to be on SSDI over time than those with OPD (RRR = 1.18, 95% CI 1.13, 1.23, *p* < 0.001).

**Fig. 2:**
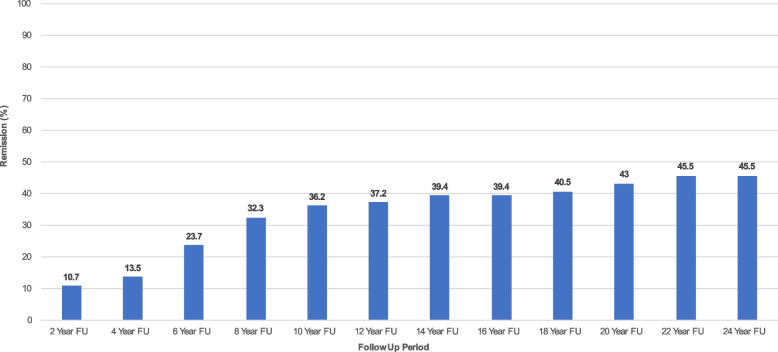
Remission of SSDI utilization among patients with borderline personality disorder over 24 years of prospective follow-up (*N* = 290). *Note.* Remission is defined as no longer being on SSDI at any given follow-up period after having been on SSDI at baseline. Each follow-up period displays the cumulative percentage of patients with BPD who had experienced their first remission by that time point

**Fig. 3:**
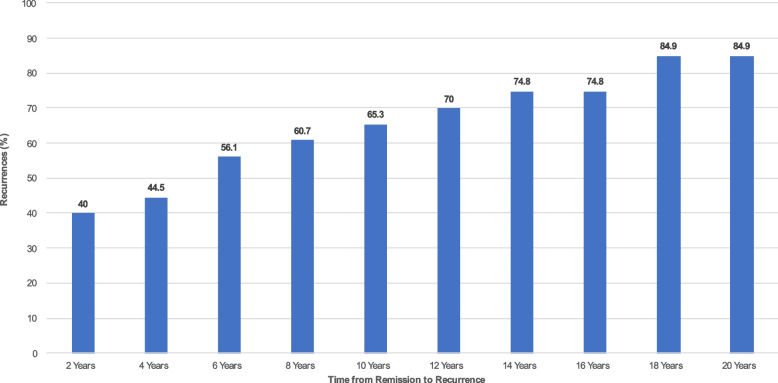
Time to recurrence of SSDI Utilization among patients with borderline personality disorder over 24 years of prospective follow-up. *Note.* A recurrence is defined as being on SSDI at any follow-up period after having a remission from SSDI utilization (i.e., patients who began the study on SSDI at baseline, went off SSDI at some point, and then went back on SSDI at a later follow-up period)

**Fig. 4:**
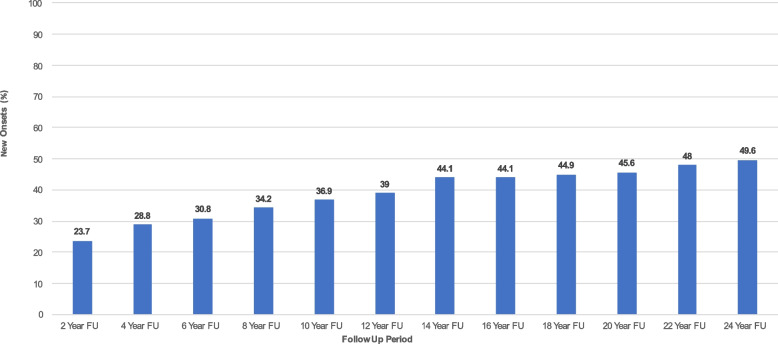
New onset of SSDI Utilization among Patients with Borderline Personality Disorder over 24 years of prospective follow-up. *Note.* New onset is defined as being on SSDI at any follow-up period after not having been on SSDI at baseline. Each follow-up period displays the cumulative percentage of patients with BPD who had experienced a new onset by that time point

Figures 2, 3, 4 focus on the survival analyses in BPD. Fig. 2 describes the cumulative percentage of individuals with BPD who had experienced their first remission from SSDI utilization by a specific timepoint. For example, at 2-year follow-up, only approximately 11% of patients with BPD who were on SSDI at baseline experienced such a remission. However, by 24-year follow-up, 46% of patients with BPD are able to get off SSDI for at least one follow-up period over the entire timespan.

Figure 3 depicts time to recurrence rates of SSDI. These denote rates of recurrence that occurred two years after remission from SSDI, 4 years after remission from SSDI, 6 years after remission from SSDI utilization, and so forth. From Fig. 2, it became clear that by 24 years of follow-up, approximately 46% of patients with BPD experienced a remission from SSDI at some point during the study; of this 46% of patients, approximately 85% (according to Fig. 3 far right) experienced a recurrence of SSDI utilization by the end of the study.

Figure 4 displays the cumulative percentages of new onsets of SSDI utilization over the entire study period. For instance, at 2-year follow-up, approximately 24% of patients with BPD who had not been on SSDI at baseline were now on SSDI. By 24-year follow-up, approximately 50% of patients with BPD experienced a new onset of SSDI utilization.

Focusing on BPD, Table 1 presents the bivariate predictors of SSDI utilization across 24 years of follow-up (12 waves), controlling for GAF scores at intake. In total, 16 of the 28 variables examined were found to be significant in these bivariate analyses. These variables were: older age, non-white race, presence of prior psychiatric hospitalizations, history of sexual abuse, more severe childhood abuse of a verbal/emotional/physical nature, more severe childhood neglect, lower childhood competence, lower parental competence, lower IQ, presence of lifetime PTSD, presence of any anxiety disorder, presence of adult rape, presence of physically abusive partner, and three facets of normal personality (higher neuroticism, lower openness to experience, and lower agreeableness). Non-significant variables in the model included sex, age of onset of symptoms, age of first treatment, presence of early childhood separations, presence of parental divorce, number of positive childhood relationships, presence of ADHD, presence of a mood disorder, presence of a substance use disorder, presence of an eating disorder, extraversion, and conscientiousness.

**Table 1:** Bivariate baseline predictors of Social Security Disability Insurance (SSDI) over 24 years of prospective follow-up

	% (N)	Mean (SD)	RRR	Z	*P*	95% CI
**Demographic Characteristics**						
** 26 Years Old or Older at Index Admission (median age = 26)**	44.48(129)		1.26	4.91	** < .001**	1.14, 1.38
Gender (Male)	19.66(57)		0.96	-0.62	.532	0.56, 1.08
** Race (Non-White)**	12.76(37)		1.13	2.01	**.045**	1.00, 1.28
**Treatment History**						
Age of Onset of Symptoms		10.83(5.28)	1.00	-0.57	.571	0.99, 1.01
Age of First Treatment		17.26(6.18)	1.00	0.70	.486	1.00, 1.01
** Presence of Prior Hospitalizations**	78.62(228)		1.26	4.35	** < .001**	1.13, 1.39
**Pathological Childhood Experiences**						
** Presence of Sexual Abuse**	62.41(181)		1.04	3.94	** < .001**	1.02, 1.06
** Severity of Other Forms of Abuse**		7.28(5.34)	1.08	3.71	** < .001**	1.04, 1.13
** Severity of Neglect**		14.68(10.66)	1.01	3.73	** < .001**	1.00, 1.01
Presence of Early Childhood Separations	34.48(100)		1.00	0.57	.568	0.99, 1.02
Presence of Parental Divorce	40.00(116)		1.02	0.40	.689	0.93, 1.12
**Childhood Experiences/Risk Factors**						
** Lower Childhood Competence**		7.63(3.96)	0.99	-2.34	**.019**	0.99, 1.00
Number of Positive Relationships		7.23(4.11)	0.99	-1.82	.069	0.98, 1.00
** Lower Parental Competence**		17.67(7.29)	0.99	-2.71	**.007**	0.97, 1.00
** Lower IQ**		104.16(11.96)	0.99	-8.13	** < .001**	0.98, 0.99
Presence of Attention Deficit Hyperactivity Disorder	27.59(80)		1.09	1.75	.081	0.99, 1.21
**Lifetime Axis I Disorders**						
History of Mood Disorder	96.90(381)		1.10	0.90	.367	0.89, 1.37
History of Substance Use Disorder	62.07(108)		1.00	-0.01	.993	0.91, 1.10
** History of PTSD**	58.28(169)		1.22	4.16	** < .001**	1.11, 1.34
** History of Another Anxiety Disorder**	69.31(201)		1.11	2.24	**.025**	1.01, 2.23
History of Eating Disorder	53.79(156)		1.08	1.69	.090	0.99, 1.18
**Aspects of Temperament**						
** Neuroticism**		35.08(7.02)	1.01	2.65	**.008**	1.00, 1.01
Extraversion		22.59(6.97)	0.99	-1.52	.127	0.99, 1.00
** Openness**		29.81(6.62)	0.99	-2.82	**.005**	0.98, 1.00
** Agreeableness**		30.35(6.72)	0.99	-2.38	**.017**	0.99, 1.00
Conscientiousness		28.56(7.77)	1.00	1.15	.251	1.00, 1.01
**Adult Adversity**						
** Presence of Adult Rape History**	31.38(91)		1.14	2.74	**.006**	1.04, 1.26
** Presence of Physically Violent Partner**	71.72(208)		1.18	3.09	**.002**	1.06, 1.31

Predicted was the course (presence per wave) of utilization of SSDI. Age: binary variable (older than 26 years at baseline). Hospitalization: Number of prior hospitalizations at baseline. Severity Abuse: Severity of emotional, verbal, and physical abuse

*IQ* Lower level of intelligence at baseline, *PTSD* Presence of DSM-III-R diagnosis of Post-Traumatic Stress Disorder, *SSDI* Social Security Disability Insurance. Analysis controlled for Global Assessment of Functioning at baseline

Table 2 shows the significant multivariate predictors of SSDI over time. As outlined above, baseline GAF score was included in these analyses to control for overall baseline severity. The remaining four significant predictors were: older age, more severe Childhood abuse of a verbal/emotional/physical nature, lower IQ, and the presence of a lifetime co-morbid PTSD diagnosis at baseline.

**Table 2:** Significant multivariate predictors of Social Security Disability Insurance (SSDI) over 24 years of prospective follow-up

Predictors	RRR	SE	Z	*P*	95% CI
Age 26 or older	1.19	0.05	4.07	< .001	1.10, 1.30
Severity of non-sexual Abuse	1.01	0.00	2.86	.004	1.00, 1.02
Lower IQ	0.99	0.00	-8.16	< .001	0.98, 0.99
Baseline history of PTSD	0.89	0.04	-2.80	.001	0.81, 0.99

Predicted was the course (presence per wave) of utilization of SSDI. Age: binary variable (older than 26 years at baseline). Severity Abuse: Severity of emotional, verbal, and physical abuse

*IQ* Lower level of intelligence at baseline, *PTSD* Presence of DSM-III-R diagnosis of Post-Traumatic Stress Disorder at baseline. Analysis controlled for Global Assessment of Functioning at baseline

## Discussion

The present study has four main results. It documents that, despite some fluctuations, the utilization of SSDI over 24 years is remarkably stable in a study population of patients with borderline personality disorder, with prevalence rates ranging between 40.7% (at baseline) and 51.9% (at 24-year follow-up) and a mean of 47.2% (arithmetic mean over the 13 waves). It is noteworthy that these numbers are roughly three times higher than in the study population with other (non-borderline) PDs which is consistent with earlier results showing that rates of SSDI utilization by people with BPD vary between 41 and 52%, and for OPD between 8 and 19% [41]. This observation suggests that the functional impairment in patients with BPD may be more severe than in patients with other personality disorders. On this group level analysis, a high stability in the usage of SSDI may be noted. This result may reflect that the disorder itself interferes with functioning well from an occupational viewpoint, and with the capacity to build a life worth living [10].

The second main result indicates that on a within-person—or individual—level of analysis, consistent with observations on the course of psychosocial functioning in BPD [36], SSDI usage may be fluctuating significantly over a quarter of a century. Despite the observation that approximately half of this BPD sample at each time point was self-supporting, a strong fluidity may be found on the individual level. Therefore, a more nuanced view is warranted here: over a quarter of a century, 46% get off SSDI out of which 85% get back on again, with a total of new onsets of one out of two.

The third result suggests such specific baseline predictors that foreshadow the utilization of SSDI over time in patients with BPD. The bivariate analyses highlighted that specific demographic variables, childhood experiences, lifetime psychiatric diagnoses and temperament may be relevant. Among the demographic characteristics, non-white race and being older than 26 are factors increasing the likelihood of being on SSDI. Among variables related with pathological and other childhood experiences, a history of sexual abuse, the severity of other abuse and of neglect, a lower childhood and parental competence, along with a lower IQ, predicted SSDI over time. Among lifetime psychiatric diagnoses, post-traumatic stress disorder and an anxiety disorder increased the likelihood of SSDI over time, along with aspects of adult adversity (presence of adult rape and of a physically violent partner). Finally, aspects of temperament predicted the likelihood of SSDI over time, in the sense of higher neuroticism and lower openness to experience and lower agreeableness in patients who eventually will be on SSDI. These results indicate a potential for psychosocial and contextual vulnerability in patients with BPD, in addition to the specific severity of symptoms [36]. This vulnerability may interfere with good adjustment and with the fact of being self-supportive of their basic needs via a regular occupation.

The fourth result synthesizes the three most important factors related to SSDI in BPD in the multi-variate analyses: being older at initial hospitalization, lifetime adversity, and the absence of protective factors contribute together to the use of SSDI. Absence of protective factors seems an important predictor, as in a comparison between adult patients with BPD, adolescent patients with BPD and healthy adolescent controls, Borkum et al. [3] found that the former had the smallest number of protective factors (and the latter the highest). Absence of such objective protective factors (i.e., lower level of intelligence) may indicate particular vulnerability to long-term psychosocial problems in BPD [36],for adolescents with BPD, see [3, 13]. For adult patients with BPD, we hypothesize that patients with lower IQ, and greater lack of protective factors may have it more difficult to obtain or to maintain an educational or occupational adjustment of which he or she can be proud. Similarly, patients who present with a lifetime diagnosis of PTSD and who had more severe non-sexual abuse in childhood were more likely to become a recipient of SSDI, which may be explained by adjustment-interfering problems related with shame or depersonalization as a consequence of the traumatic past [11, 34]. Patients who are older at index hospitalization are more likely to be recipients of SSDI over time. Older age may indicate that the patient may have struggled for a sustained period, which may have led his or her treatment team to support applying for SSDI to give that person a small, but ongoing, income, as well as access to medical and/or psychiatric care.

Of note, in the multivariate analyses, factors of temperament did not meet the threshold for statistical significance. Contrary to earlier discussions [36], a patient’s personality traits were unrelated to receiving SSDI. This result indicates that contributions of personality aspects – the notion of not fitting into society, having a specific temperament or presenting interpersonal problems [41] – seem less important when predicting SSDI over time, however, vulnerability resulting from lifetime adversity *is* important.

Interestingly, several relevant predictors did not emerge from our bivariate nor multivariate analyses. For example, among the variables denoting treatment history, both age of onset of symptoms and the age of first treatment do not predict SSDI over time. Treatments may have a verified impact on the course of symptoms in BPD, but do not seem to directly impact the capacity to hold a self-supportive occupation nor the utilization of SSDI over time. Clinicians could in some cases more explicitly focus on how to foster these self-supportive skills and capacities through treatment, possibly preventing further course of illness and SSDI status over time. In other cases, clinicians could help patients on SSDI, or in need of SSDI, to promote acceptance of a situation that may not have been seen as desirable by the patient (or the society).

While sexual and other abuse, and the severity of neglect, predict SSDI status over time on a bivariate level, childhood separations and parental divorce did not. This may indicate the impact of *severity* of pathological childhood experiences on the further course of illness and SSDI status, which is confirmed by our multivariate analysis. It is also interesting to note that the number of positive childhood relationships did not impact negatively – nor positively – the course of SSDI over time. Finally, among the psychiatric diagnoses analysed, only the presence of PTSD was significant in our multivariate analyses as a predictor of SSDI over time. This makes sense in line with the above and in the context of the impact of traumatic experiences and their pathological consequences on functioning [8, 27]. The presence of life-time PTSD may have an impact on the long-term psychosocial consequences, via the lower capacity to self-support (SSDI usage). Further research should examine the long-term impact of SSDI usage on psychosocial functioning over time. It seems important to understand whether long-term usage of SSDI may mediate the link between life-time PTSD and psychosocial adaptation. Essentially, we speculate that the fluctuating and more dynamic usage of SSDI may function as a protective factor between PTSD and poor psychosocial adaptation.

The clinical implications of the findings are twofold. First, clinicians should carefully assess the main risk factors potentially contributing to SSDI status over time. Ideally, this should be done as early as possible, offering a timely window of opportunity for prevention and early intervention which is tailored to the individual’s symptom profile and severity, occupation status and history of SSDI. Second, based on such an assessment, clinicians may a) decide to support SSDI for a particular patient, or b) decide to take direct action to prevent a patient from becoming a recipient of SSDI. Both options may involve proposing additional help by a social worker or occupational therapist, building career-supporting resources, and encouraging vocational rehabilitation programs. Specific vocational rehabilitation programs have been developed in the domain of mental health, and for individuals with BPD in particular [15], with highly promising results [9]. Some protective factors related to building long-term resilience could be a central part of treatment. For example, clinicians may consider integrating a systematic enhancement of malleable protective factors into the treatment plan of those patients who lack them. It may be helpful to consider teaching the patient specific emotion regulation and interpersonal skills (e.g., DEAR MAN fostering self-assertiveness in the patient), within dialectical behavioral therapy [12, 17] and by using specific trauma-informed treatment for those patients with BPD and co-occurring PTSD [2].

A number of limitations need to be acknowledged. All recruited patients were inpatients at the time of recruitment. The results may not generalize to less severely ill individuals. Finally, we need to acknowledge that the actual motivation for being on SSDI remains unclear for the present sample and may change over time; for example, someone may initially have been on SSDI for psychiatric reasons, gotten off it as their mental health and functioning improved, and then got on it again because he or she developed a chronic medical condition that prevented from working.

## Conclusions

The present study highlighted the relative stability over a 24-year follow-up of social security disability insurance (SSDI) in a population of patients with borderline personality disorder. On an individual level, this study also highlighted the fluidity of being on SSDI. Finally, the results of this study suggest that a combination of demographic factors, childhood adversity, natural endowment and comorbidity are significant predictors of receiving SSDI over 24 years of prospective follow-up.

## Data Availability

Data are available upon request from the last author.
